# Clinical Factors Associated with Arterial Stiffness in Chronic Kidney Disease

**DOI:** 10.3390/jcm12031077

**Published:** 2023-01-30

**Authors:** Jin Yao, Zheyi Dong, Qian Wang, Zhe Li, Weiguang Zhang, Wenwen Lin, Yayong Luo, Hangtian Li, Xinru Guo, Li Zhang, Guangyan Cai, Wanjun Shen, Shuwei Duan, Xiangmei Chen

**Affiliations:** 1 School of Clinical Medicine, Guangdong Pharmaceutical University, Guangzhou 510006, China; 2 Department of Nephrology, First Medical Center of Chinese PLA General Hospital, Nephrology Institute of the Chinese People’s Liberation Army, State Key Laboratory of Kidney Diseases, National Clinical Research Center for Kidney Diseases, Beijing Key Laboratory of Kidney Disease Research, Beijing 100853, China; 3 The First Affiliated Hospital, College of Clinical Medicine, Henan University of Science and Technology, Luoyang 471003, China

**Keywords:** chronic kidney disease, arterial stiffness, augmentation index

## Abstract

Background: Arterial stiffness influences the prognosis of patients with end-stage kidney disease; however, the factors that promote arterial stiffness in chronic kidney disease (CKD) patients remain unknown. We aimed to explore the clinical factors associated with arterial stiffness in CKD. Methods: Between September 2017 and September 2022, all CKD patients treated at the Department of Nephrology, General Hospital of the Chinese People’s Liberation Army, excluding dialysis patients, were screened and their medical records within the last month were collected. Arterial stiffness was measured by the augmentation index (AIx). The correlative clinical factors with arterial stiffness were explored in different linear regression models. Results: 559 patients were included in the study. AIx@75 increased as the deterioration of CKDG1–CKDG5, with values of 1 (−9, 11), 5.5 (−4, 13.25), 9 (0, 16), 12 (1.5, 23.5), and 22 (13, 28), respectively (Z = 63.03, *p* < 0.001). Multivariate linear regression analysis showed that AIx@75 was positively associated with female sex (β = 8.926, 95% confidence interval (CI) 6.291, 11.562, *p* < 0.001), age (β = 0. 485, 95% CI 0.39, 0.58, *p* < 0.001), mean arterial pressure (MAP) (β = 0.255, 95% CI 0.159, 0.35, *p* < 0.001), and was negatively associated with ACEI/ARB (β = −4.466, 95% CI −6.963, −1.969, *p* < 0.001) and glucocorticoid (β = −3.163, 95% CI −6.143, −0.183, *p* = 0.038). Smoking, eGFR, hemoglobin, and cause of disease were associated with AIx@75 in multivariate linear regression models when considering factors partly. Conclusions: Female, age, smoking, MAP, eGFR, cause of disease, ACEI/ARB, and glucocorticoid were found to be associated with atherosclerosis in CKD patients.

## 1. Introduction

Chronic kidney disease (CKD) is a global public health problem [1], representing a direct cause of morbidity and mortality worldwide and an important risk factor for cardiovascular disease [2], especially at the end-stage of renal disease (ESRD). Arterial stiffness is a major factor in the increased cardiovascular risk of CKD and also accelerates the progression of CKD. In the general population, risk factors for atherosclerosis include aging, smoking, hypertension, diabetes mellitus, dyslipidemia, and hyperuricemia, among which hypertension, diabetes mellitus and hyperuricemia also contribute to kidney damage and the development of CKD [3].

Previous studies suggested that with the decrease in eGFR, the degree of arteriosclerosis worsens, and the comorbidity or complication such as diabetes mellitus, hypertension, and hyperuricemia are also common in patients with CKD [4,5,6,7]. However, the risk factors of arteriosclerosis in CKD patients are still unclear. This study aims to find out the related factors of arteriosclerosis in CKD patients through cross-sectional investigation as a basis for further study of its risk factors.

## 2. Materials and Methods

### 2.1. Participants

This single-center cross-sectional study was conducted from September 2017 to September 2022 on adults with CKD who were hospitalized at the General Hospital of the Chinese People’s Liberation Army, excluding patients on dialysis. The inclusion criteria were as follows: (i) age >18 years, (ii) CKD was diagnosed in line with the 2012 KDIGO (Kidney Disease: Improving Global Outcomes) guidelines by the investigators prior to enrollment [6]. The exclusion criteria were as follows: (i) incomplete medical history or clinical examination results; (ii) hemodialysis; and (iii) patients who were unsuitable for evaluation of the Endo-PAT, such as patients who had wounds on their fingers, Raynaud’s phenomenon, arteriovenous fistulas, and other instability factors that the investigators felt made the patient unsuitable for participation.

This study was approved by the Ethics Committee of the General Hospital of the Chinese People’s Liberation Army (No. S2017-133-01). The study was conducted in accordance with the Declaration of Helsinki and all participants were enrolled in the study after providing written informed consent.

### 2.2. General Information Collection

The information collected included sociodemographic characteristics, medical history, laboratory tests and medication history within the last month. Practicing physicians who were trained in Good Clinical Practice (GCP) collected the above data through a bedside inquiry and medical record system. If there are multiple different records of the same indicator within a month, the results closest to the AIx measure date were recorded. The augmentation index (AIx) was measured using an Endo-PAT 2000 device (Itamar Medical, Caesarea, Israel) by researchers following standard protocols.

### 2.3. Laboratory Testing

The subjects were required to fast for 8–12 h before blood sample collection. The following blood biochemical parameters were measured in the blood samples using standard assays: alanine aminotransferase (ALT), aspartate aminotransferase (AST), total protein (TP), albumin (ALB), total cholesterol (TC), triglycerides (TG), high-density lipoprotein (HDL), low-density lipoprotein (LDL), fasting blood glucose (FBG), blood urea nitrogen (BUN), blood creatinine (Scr), blood uric acid (UA), serum calcium concentration(Ca), serum phosphorus concentration (P), potassium (K), serum sodium concentration (Na), serum magnesium concentration (Mg), hemoglobin (Hb), platelet count (PLT), homocysteine (HCY), prothrombin time (PT), plasma activated partial thromboplastin time (APTT), fibrinogen (Fbg), D-dimer (D-Dimer), and C-reactive protein (CRP). The eGFR was calculated using the CKD Epidemiology Society (CKD-EPI) creatinine equation [8].

### 2.4. Assessment of Arterial Stiffness

The Endo-PAT 2000 device (Itamar Medical, Caesarea, Israel) was used to assess the AIx, representing arterial stiffness. All patients underwent testing at a temperature of 21–24 °C in a dimly lit and quiet environment by an investigator with professional training and at least one year of experience according to the operating manual [9]. All patients were placed in the supine position and were forbidden to smoke or exercise for at least 3 h prior to the test. The occluded cuff was placed on one upper arm and a finger probe was placed on the index finger of each hand to record the signal. The procedure included a 5-min baseline, 5-min occlusion, and 5-min post-occlusion signal recording. The occlusion pressure should be 60 mmHg higher than the systolic pressure measured before the test, or 200 mmHg; then, complete occlusion was judged by the device. The AIx and AIx@75, the standard AIx when the calculation was corrected to 75 heart beats per min, values were automatically generated.

### 2.5. Statistical Analysis

All statistical analyses were performed using IBM SPSS 26.0 software. Numerical variables are expressed as the mean ± standard deviation or median (IQR) based on distribution. Categorical variables were expressed as frequencies (%). The demographic, clinical, and laboratory tests were compared between groups according to the staging of CKD using one-way ANOVA, Kruskal–Wallis test, and Pearson χ^2^ test. AIx and AIx@75 were used to establish univariate linear regression models to screen the relationship between arterial stiffness and other variables in Chinese patients with CKD. Then, AIx@75 was used to explore the dependent variables in multivariate linear regression analysis. *p* < 0.05 was regarded as statistically significant.

## 3. Results

### 3.1. Baseline Characteristics

**Figure 1:**
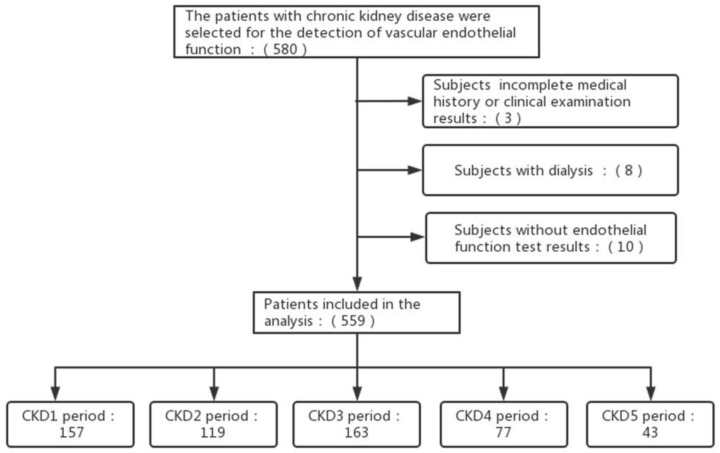
Flowchart of participant screening. Abbreviations: CKD: chronic kidney diseases.

A total of 559 participants were eligible for inclusion. Figure 1 showed a flowchart of participant screening. The baseline characteristics of the patients were summarized in Table 1 and Table 2. Besides AIx and AIx@75, age, mean arterial pressure, urinary protein, TP, BUN, UA, TG, P, K, Mg, Fbg, HCY, D-Dimer, and the proportion of patients with hypertension, diabetes mellitus, hyperuricemia, and coronary heart disease (CHD) increased with the decrease of eGFR (*p* < 0.05). The levels of ALT, TC, HDL, Hb, PLT, and the proportion of ACEI/ARB, immunosuppressant, and glucocorticoid drug use were decreased with the decrease in eGFR (*p* < 0.05). More immune nephropathy patients, such as those undergoingIgA nephropathy, showed normal kidney function, while more metabolic nephropathy, such as diabetic nephropathy, with injured kidney function, and at the same time, some patients with both or unidentified.

### 3.2. Univariate Linear Regression Analysis of Factors Related to Arterial Stiffness

Univariate linear regression was used to analyze the related factors of AIx and AIx@75 in patients with CKD (Table 3). Age, female sex, MAP, hypertension, diabetes mellitus, CHD, metabolic nephropathy, metabolic and immune combined nephropathy, intrarenal arteriosclerosis, FBG, BUN, Scr, K, Mg, and use of statins were positively associated with AIx and AIx@75 (*p* < 0.05). At the same time, eGFR, ALT, Hb, and use of ACEI/ARB, immunosuppressant, and glucocorticoid were negative associated with Aix and Aix@75 (*p* < 0.05). Considering the correlative factors with Aix and Aix@75 were consistent, and AIx@75 was adjusted the influence of heartbeats, AIx@75 was used to explore the correlation factors in multivariate linear regression analysis. After collinearity diagnostics, potential relative factors with AIx@75 including female, age, diabetes mellitus, intrarenal arteriosclerosis, smoking, CHD, MAP, ALT, AST, eGFR, Hb, P, Mg, K, Fbg, TC, TG, HDL, immunosuppressant, glucocorticoid, statins, and ACEI/ARB were included in different models of multivariate linear regression analysis, respectively.

### 3.3. Correlation Factors of the Reflection Enhancement Index AIx@75 in Patients with CKD

Considering too many factors should be included in the regression analysis, five multivariate linear regression models on various levels were developed to explore the correlative factors with AIx@75. Model 1 was based on general patient information as independent variables, including female sex, age, smoking, MAP, diabetes, CHD, and intrarenal atherosclerosis. Female sex, age, MAP, and smoking were found positively correlated with AIx@75 in Model 1 (Table 4). Model 2 was based on laboratory tests as independent variables, including eGFR, ALT, AST, P, K, Mg, Hb, Fbg, TC, TG, and HDL. Only Hb and eGFR were independently associated with AIx@75 in Model 2 (Table 4). Model 3 was built based on Models 1 and 2 as independent variables, including female sex, age, smoking, MAP, diabetes mellitus, CHD, intrarenal arteriosclerosis, eGFR, ALT, AST, P, K, Mg, Hb, Fbg, TC, TG, and HDL. After adjusted for female and age, MAP and eGFR were correlated with AIx@75 in Model 3 (Table 4), verify clear whether the cause of disease was related to arteriosclerosis, Model 4 was developed based on Model 3 plus cause of disease as independent variables. After being adjusted for female sex, age and MAP, eGFR and cause of disease were independently associated with AIx@75 in Model 4 (Table 4). Compared with immune nephropathy, metabolic nephropathy were borderline associated with AIx@75, while metabolic and immune combined nephropathy were significant correlated with AIx@75. Then, Model 5 was built based on Model 4 to find out if the therapeutic drug was correlated with AIx@75. Model 5 was based on Model 4 plus therapeutic drug as independent variables. After being adjusted for female sex, age, and MAP, use of ACEI/ARB and glucocorticoid were negatively correlated with AIx@75; at the same time, neither eGFR nor cause of disease were correlated with AIx@75 (Table 4).

## 4. Discussion

In this study, we investigated the relationship between AIx@75 and various clinical factors in CKD. In addition to age and female, MAP, eGFR, and the cause of disease were independently associated with arteriosclerosis in CKD. The use of ACEI/ARB and glucocorticoid could change the association between eGFR and the cause of disease with AIx.

Arteriosclerosis is an important factor of poor prognosis in end-stage CKD patients. Numerous studies show that CKD progression and atheromatosis progression are closely associated [10,11].

Studies had shown that central pulse pressure (CAP), AIx, pulse wave velocity (PWV), and carotid and femoral arterial ultrasound were powerful tools for predicting atheromatosis progression in patients with CKD [10,12,13]

In this study, we selected the AIx as noninvasive assessment indicators for arterial stiffness because of its simplicity of operation and portability.

The present study found that in glucocorticoid use, Hb was negatively associated with atherosclerosis and etiology was associated with atherosclerosis, unlike previous studies. We found the use of glucocorticoid were negatively associated with atherosclerosis in CKD patients and could change the association between eGFR and the cause of disease with atherosclerosis. So, we considered glucocorticoid maybe decrease atherosclerosis by improving renal function, especially in patients with immune inflammation. The impact of glucocorticoids on atherosclerosis was unclear [14]. Clinical and preclinical studies have shown both atheroprotective and proatherogenic responses to glucocorticoids, so effects depend upon their multifactorial actions [15]. Long-term use of glucocorticoids can cause high blood pressure and arteriosclerosis due to water and sodium retention and elevated blood lipids. In the study, half of the patients had no impaired renal function, while the blood pressure and blood lipids of most patients were controlled well. All of these decreased the side effect of glucocorticoid, so glucocorticoid showed help to reduce atherosclerosis.

Hb showed a negative correlation with arterial stiffness in the study, while Hb was positively correlated with arterial stiffness in healthy people [16,17,18,19]. The difference may be caused by the different study population. High Hb in healthy people was a symptom of chronic hypoxia, so atherosclerosis worsens with the increase in Hb. The proportion of CKD patients with injured kidney function (50.6%) and anemia (22.9%) were high in this study. Anemia and hypertension are both common complications of CKD, especially in patients with injured kidney function and the degree of anemia increases with the aggravation of renal impairment. Both eGFR and Hb were correlated with the atherosclerosis when only considering laboratory testing. After adjustment for age, female, eGFR and MAP, Hb was no longer an independent correlation factor for atherosclerosis. Thus, we think the correlation between Hb and atherosclerosis in CKD may be influenced by renal function and blood pressure. More specialized studies are needed to further evaluate this.

Although all of the metabolic relative factors such as diabetes mellitus, hyperlipidemia, and hyperuricemia were not correlated with atherosclerosis, cause of disease was related with atherosclerosis in our study. Patients with metabolic were higher degree of atherosclerosis than patients with immune nephropathy. Elevated glucose level, dyslipidemia, and other metabolic alterations were tightly involved in the almost every step of the atherogenic process [20]. It suggested that CKD, like diabetes and dyslipidemia, was a risk factor for atherosclerosis. The other reason was the drug therapy. In fact, among the patients included in this study, the proportion of lipid-lowering drugs (49.9%) and hypoglycemic drugs (53.8%) are not low. After adjusting eGFR, cause of disease was related with atherosclerosis in our study. It inferred CKD and metabolic alterations such as diabetes and dyslipidemia had synergistic effect on atherosclerosis. After adjusting for drug therapy, cause of disease was no longer independent factors associated with atherosclerosis, indicating that clinical treatment can affect the correlation between cause of disease and atherosclerosis.

As in previous studies, atherosclerosis was more severe in females, and eGFR was negatively associated with atherosclerosis. Our study found that female patients had more severe arteriosclerosis than male patients in CKD. Previous studies on healthy people suggest that estrogen has an inhibitory effect on atherosclerosis. The degree of arteriosclerosis in female before menopause was lower than that in men of the same age, while there was no gender difference in arteriosclerosis after menopause [21]. The average age (49.4) of enrolled patients was older and the proportion of patients with hypertension (69.7%) was higher. Female adapt to pressure overload differently from men, with more prominent remodeling of a concentric rather than eccentric nature, and a more profound response to hypertension and obesity than men [22]. A study demonstrated that female patients had higher AIx than male patients in both predialysis and dialysis patients [23], and that females were more susceptible to arterial aging [24,25]. Our findings were consistent with these findings. This phenomenon was partly due to their lower height and the closer distance between the heart and the reflex site [26,27,28]. It has also been reported that these results were related to the thinner diameter of the radial artery and the lower central arterial pulse pressure in females compared to men [28]. Therefore, more attention should be paid to the evaluation of arteriosclerosis in females with CKD.

Our study found that arteriosclerosis was negatively correlated with eGFR, which was consistent with previous findings [23]. It was considered that with the decrease in eGFR, the body’s ability to clear large and small molecular metabolites reduced incidence of vascular damage. At the same time, arteriosclerosis can also promote the progression of CKD by affecting microcirculation [29].

Similar to the general population, atheromatosis progression in CKD is more prevalent among smokers, high levels of MAP, and older patients [10,12], and our research was consistent with that. ACEI/ARB with antiproteinuric effect may decrease the risk of atherosclerosis [30], our finding in the CKD population was consistent with this.

Some limitations should be noted. First, as this is a cross-sectional design, it is difficult to determine the causal relationship between these influences and arterial stiffness. Second, our data came from a single center, which resulted in some selection bias. Third, AIX@75 is a measure of the reflected wave of the terminal artery. However, other indicators of arterial stiffness obtained through the brachial or ankle artery may lead to heterogeneity in arterial stiffness and result in bias.

## 5. Conclusions

In conclusion, Female, age, smoking, MAP, eGFR, cause of disease, ACEI/ARB, and glucocorticoid were found to be associated with atherosclerosis in CKD.

## Figures and Tables

**Table 1:** Demographic characteristics of the participants.

Variable	Total (N = 559)	CKD1 Period (N = 157)	CKD2 Period (N = 119)	CKD3 Period (N = 163)	CKD4 Period (N = 77)	CKD5 Period (N = 43)	Statistics	*p* Value
Female, n (%)	171 (30.6)	53 (33.8)	28 (23.5)	60 (36.8)	18 (23.4)	1 2(27.9)	8.539 **	0.074
Age (years)	52 (39, 59)	45 (33,54)	51 (38.75, 58.25)	55 (44, 61)	56 (46, 62)	56 (51, 63)	54.881 ***	<0.001
BMI (kg/m^2^)	25.4 (22.83, 27.67)	25.1 (22.4, 27.9)	25.3 (22.8, 27.6)	25.5 (22.8, 28.1)	25.6 (23.3, 27.5)	25.3 (22.5, 27.3)	0.799 ***	0.939
BMI							15.162 **	0.513
BMI < 18.5	10 (1.8)	0	5 (0.9)	2 (0.4)	2 (0.4)	1 (0.2)	-	-
18.5 ≤ BMI < 24	184 (32.9)	57 (10.2)	38 (6.8)	52 (9.3)	21 (3.8)	16 (2.9)	-	-
24 ≤ BMI < 28	235 (42.1)	62 (11.1)	48 (8.6)	66 (11.8)	40 (7.2)	19 (3.4)	-	-
28 ≤ BMI < 30	65 (11.6)	17 (3)	14 (2.5)	21 (3.8)	9 (1.6)	4 (0.7)	-	-
30 ≤ BMI < 40	65 (11.6)	21 (3.8)	14 (2.5)	22 (3.9)	5 (0.9)	3 (0.5)	-	-
MAP (mmHg)	97 (89.67, 106.58)	95 (86.7, 101.7)	97 (89.6, 106.4)	97 (88.8, 105.3)	102 (93, 109.5)	10 1(95, 112)	23.272 ***	<0.001
Smoking, n (%)	149 (26.7)	38 (24.2)	33 (27.7)	41 (25.2)	26 (33.8)	11 (25.6)	2.758 **	0.599
Drinking, n (%)	132 (23.6)	37 (23.6)	30 (25.2)	34 (20.9)	22 (28.6)	9 (20.9)	2.075 **	0.722
Hypertension, n (%)	389 (69.7)	77 (49)	72 (60.5)	125 (76.7)	74(96.1)	41 (95.3)	78.896 **	<0.001
Diabetes mellitus, n (%)	301 (53.8)	65 (41.4)	58 (48.7)	88 (54)	55 (71.4)	35 (81.4)	33.744 **	<0.001
CHD, n (%)	58 (10.4)	8 (5.1)	13 (10.9)	16 (9.8)	11 (14.3)	10 (23.3)	13.738 **	0.008
Hyperuricemia, n (%)	234 (41.9)	43 (27.4)	41 (34.5)	83 (50.9)	40 (51.9)	27 (62.8)	32.65 **	<0.001
Cause of disease							102.456 **	<0.001
Immune nephropathy	283 (50.6)	110 (19.7)	71 (12.7)	75 (13.4)	21 (3.8)	6 (1.1)	-	-
Metabolic nephropathy	171 (30.6)	16 (2.9)	24 (4.3)	58 (10.4)	42 (7.5)	31 (5.5)	-	-
Both	28 (5)	9 (1.6)	6 (1.1)	10 (1.8)	2 (0.4)	1 (0.2)	-	-
Unidentified	77 (13.8)	22 (3.9)	18 (3.2)	22 (3.9)	12 (2.1)	5 (0.9)	-	-
Intrarenal arteriosclerosis, n (%)	240 (42.9)	64 (40.8)	53 (44.5)	88 (54)	27 (35.1)	8 (18.6)	41.791 **	<0.001
Endo-PAT 2000Parameters								
AIx	8.20 ± 18.47	1.54 ± 18.079	5.34 ± 18.221	10.27 ± 17.347	13.73 ± 17.275	22.33 ± 15.788	15.750 *	<0.001
AIx@75	7 (−3, 16)	1 (−9, 11)	5.5 (−4, 13.25)	9 (0, 16)	12 (1.5, 23.5)	22 (13, 28)	63.03 ***	<0.001
Medications								
ACEI/ARB, n (%)	353 (63.3)	129 (82.2)	91 (76.5)	112 (68.7)	19 (24.7)	3 (7)	143.205 **	<0.001
Antiplatelet, n (%)	136 (24.4)	35 (22.3)	25 (21)	46 (28.2)	24 (31.2)	6 (14)	6.878 **	0.142
Statins, n (%)	279 (49.9)	86 (54.8)	56 (47.1)	76 (46.6)	45 (58.4)	16 (37.2)	7.594 **	0.108
Immunosuppressant, n (%)	170 (30.5)	72 (45.9)	40 (33.6)	43 (26.4)	13 (16.9)	2 (4.7)	39.675 **	<0.001
Glucocorticoid, n (%)	118 (21.1)	47 (29.9)	27 (22.7)	32 (19.6)	9 (11.7)	3 (7)	16.999 **	0.002

Note: categorical variables are expressed as numbers (percentages) and continuous variables are expressed as mean standard deviation or median (interquartile spacing). * ANOVA; ** Chi-square test; *** Kruscal–Wallis test. Abbreviations: ACEI: angiotensin-converting enzyme inhibitor; AIx: arterial reflection wave enhancement index; AIx@75: AIx value at a heart rate of 75 bpm; ARB: angiotensin receptor blocker; BMI: body mass index; CHD: coronary heart disease; MAP: mean arterial pressure.

**Table 2:** Clinical characteristics of the participants.

Variable	Total (N = 559)	CKD1 Period (N = 157)	CKD2 Period (N = 119)	CKD3 Period (N = 163)	CKD4 Period (N = 77)	CKD5 Period (N = 43)	Statistics	*p* Value
eGFR (mL/min/1.73 m^2^)	59.1 (33.25, 93.87)	108.1 (99.1, 119.5)	72.4 (65.1, 80.6)	43.7 (37.2, 50.4)	22.6 (19.4, 25.6)	11.3 (8.7, 13.1)	524.71 **	<0.001
Urine protein (g/day)	2.05 (0.64, 4.38)	1.9 (0.54, 4.23)	1.14 (0.50, 4.11)	1.80 (0.60, 4)	3.28 (1.16, 5.24)	3.49 (2.23, 4.96)	25.434 ***	<0.001
ALT (U/L)	14.3 (10.23, 20.10)	16.29 (11.45, 24.05)	15 (10.5, 20.95)	13.2 (9.73, 18.9)	14 (9.2, 17.75)	11.1 (8.4, 14.8)	22.624 ***	<0.001
AST (U/L)	14.05 (11.80, 17.78)	14.1 (12.1, 18.55)	14.15 (11.6, 18.78)	14.9 (12.13, 17.8)	13.7 (11.6, 16.25)	11.8 (9.1, 14.6)	20.755 ***	<0.001
TP (g/L)	61 (52.8, 66.2)	55.8 (48.2, 64.5)	61.6 (53.5, 57.1)	61.7 (54.9, 67.2)	61.9 (56.5,66)	63.9 (56.9, 69.5)	25.398 ***	<0.001
ALB (g/L)	36.5 (30.9, 40.4)	34 (26.7, 40.3)	37.8 (31.9, 41.4)	36.95 (32.1, 37)	36.3 (31.8, 39. 5)	36.5 (32.6, 39.6)	11.319 ***	0.023
FBG (mmol/L)	4.96 (4.36, 6.06)	5 (4.36, 5.8)	4.93 (4.43, 6.06)	4.85 (4.38, 5.97)	4.87 (4.16, 6.05)	5.81 (4.57, 8.39)	7.757 ***	0.101
BUN (mmol/L)	7.76 (5.48, 11.69)	4.8 (4.01, 6.01)	6.25 (5.27, 7.84)	9.18 (7.43, 10.74)	15.02 (12.32, 18)	21.74 (20.25, 27.06)	405.525 ***	<0.001
Scr (umol/L)	119.35 (81.15, 186.3)	70.2 (61.75, 78.7)	104.5 (89.88, 115.85)	146.85 (129.45, 172)	266 (232.9, 314.4)	450.3 (413.7, 555)	494.518***	<0.001
UA (umol/L)	382.25 (321.73, 453.23)	339.7 (291.05, 412.25)	364.95 (319.9, 419.63)	409.95 (340.5, 471.35)	418.7 (342, 485.8)	429.7 (354.4, 496.5)	42.64 ***	<0.001
TC (mmol/L)	4.63 (3.74, 5.50)	4.99 (4.03, 6.28)	4.42 (3.65, 5.48)	4.5 (3.68, 5.45)	4.36 (3.50, 5.18)	4.13 (3.59, 5.05)	18.851 ***	0.001
TG (mmol/L)	1.94 (1.26, 2.64)	1.80 (1.24, 2.83)	1.91 (1.27, 2.65)	1.94 (1.2, 2.63)	1.75 (1.36, 2.96)	1.95 (1.23, 2.56)	0.655 ***	0.957
HDL (mmol/L)	1.08 (0.86, 1.27)	1.17 (0.96, 1.51)	1.04 (0.85, 1.21)	1.06 (0.85, 1.26)	0.93 (0.82, 1.17)	0.93 (0.77, 1.16)	29.195 ***	<0.001
LDL (mmol/L)	2.88 (2.09, 3.53)	3.09 (2.35, 4.32)	2.59 (2.07, 3.79)	2.88 (2.08, 3.52)	2.2 (1.91, 3.09)	2.54 (1.93, 3.13)	21.111 ***	<0.001
Ca (mmol/L)	2.18 (2.07, 2.28)	2.16 (2.06, 2.26)	2.22 (2.10, 2.30)	2.2 (2.09, 2.29)	2.17 (2.09, 2.25)	2.10 (1.99, 2.15)	19.461 ***	0.001
P (mmol/L)	1.25 (1.12, 1.41)	1.22 (1.1, 1.35)	1.17 (1.08, 1.31)	1.25 (1.11, 1.36)	1.35 (1.19, 1.5)	1.65 (1.45, 1.85)	104.115 ***	<0.001
K (mmol/L)	4.02 (3.74, 4.33)	3.92 (3.65, 4.12)	3.90 (3.60, 4.18)	4.12 (3.78, 4.39)	4.25 (3.92, 4.61)	4.6 (4.25, 4.97)	94.426 ***	<0.001
Na (mmol/L)	141.4 (139.83, 142.8)	141.3 (139.8, 142.7)	141.65 (140.28, 143.2)	141.7 (140.2, 143.28)	140.6 (139, 142.6)	140.4 (139, 142.4)	15.619 ***	0.004
Mg (mmol/L)	0.85 (0.8, 0.91)	0.82 (0.77, 0.87)	0.835 (0.79, 0.89)	0.86 (0.82, 0.91)	0.9 (0.83, 0.97)	0.92 (0.83, 1)	61.037 ***	<0.001
Hb (g/L)	122.67 ± 22.78	135.11 ± 19.892	131.71 ± 19.485	118.57 ± 19.808	107.32 ± 16.414	95.56 ± 16.929	59.137 *	<0.001
PLT (10^9^/L)	221 (180, 264.75)	241(190, 285)	219 (179.5, 254.5)	210.5 (179.25, 253)	217 (177.5, 253)	208 (168, 266)	12.98 ***	0.011
HCY (umol/L)	18.2 (12.1, 20.3)	10.7(8.9, 13.8)	15.55 (11.4, 19.6)	18 (15, 22.1)	22.4 (18.5, 32.05)	26.55 (17.55, 32.92)	138.161 ***	<0.001
PT (s)	17 (16.2, 17.8)	17.3(16.6, 18.05)	17 (15.9, 17.83)	16.7 (16, 17.6)	17 (16.25, 17.8)	17.2 (16.3, 17.8)	17.317 ***	0.002
APTT (s)	34.55 (30.9, 37.8)	34.9 (30.2, 37.8)	34.6 (31.53, 38)	34.05 (31.03, 37.73)	34.3 (30.25, 37.95)	34.1 (30.9, 38.8)	0.261 ***	0.992
Fbg (g/L)	3.65 (3, 4.66)	3.47 (2.9, 4.41)	3.41 (2.76, 4.24)	3.64 (3.01, 4.52)	4 (3.27, 5.11)	4.9 (3.8, 5.6)	38.313 ***	<0.001
D-Dimer (ug/mL)	0.37 (0.22, 0.69)	0.29 (0.2, 0.6)	0.31 (0.22, 0.58)	0.35 (0.26, 0.71)	0.52 (0.35, 0.87)	0.6 (0.42, 1.06)	36.904 ***	<0.001
CRP, n (%)							42.801 **	<0.001
Tertile 0	261 (46.7)	88 (15.7)	58 (10.4)	73 (13.1)	24 (4.3)	12 (2.1)	-	-
Tertile1 (range) (0.048–0.09775)	74 (13.2)	14 (2.5)	16 (2.9)	20 (3.6)	16 (2.9)	8 (1.4)	-	-
Tertile2 (range) (0.09776–0.1)	95 (17.2)	25 (4.5)	19 (3.4)	20 (3.6)	23 (4.1)	9 (1.6)	-	-
Tertile3 (range) (0.1001–0.204)	55 (9.8)	16 (2.9)	14 (2.5)	17 (3)	5 (0.9)	3 (0.5)	-	-
Tertile4 (range) (0.20401–2.548)	73 (13.1)	14 (2.5)	9 (1.6)	30 (5.4)	9 (1.6)	11 (2)	-	-

Note: categorical variables are expressed as numbers (percentages) and continuous variables are expressed as mean standard deviation or median (interquartile spacing). * ANOVA; ** Chi- square test; *** Kruscal–Wallis test. Abbreviations: ALT: alanine aminotransferase; APTT: plasma activated partial thromboplastin time; AST: aspartate aminotransferase; ALB: albumin; BUN: blood urea nitrogen; CRP: C-reactive protein; D-Dimer: D-dimer; eGFR: estimated glomerular filtration rate; FIB: plasma fibrinogen; FBG: fasting blood glucose; Hb: hemoglobin; HCY: homocysteine; HDL: high-density lipoprotein; LDL: low-density lipoprotein; PLT: platelet count; PT: prothrombin time; Scr: blood creatinine; TC: total cholesterol; TG: triglycerides; UA: blood uric acid.

**Table 3:** Univariate linear regression analysis of factors related to arterial stiffness.

Variables	(AIx)		(AIx@75)	
	β	*p*	β	*p*
Female	6.189	<0.001	7.919	<0.001
Age (years)	0.697	<0.001	0.560	<0.001
BMI (kg/m^2^)	−0.16	0.429	−0.134	0.467
MAP (mmHg)	0.251	<0.001	0.299	<0.001
Smoking	1.55	0.382	−0.162	0.92
Drinking	0.797	0.666	−0.245	0.885
Hypertension	12.056	<0.001	8.516	<0.001
Diabetes mellitus	8.568	<0.001	8.303	<0.001
CHD	7.196	0.005	5.297	0.024
Hyperuricemia	−0.781	0.624	−1.161	0.424
Cause of disease				
Immune nephropathy	-	-	-	-
Metabolic nephropathy	10.848	<0.001	10.418	<0.001
Both	7.875	0.027	7.157	0.027
Unidentified	1.125	0.625	3.193	0.129
Intrarenal arteriosclerosis (%)	4.421	<0.001	3.563	0.001
eGFR (mL/min/1.73 m^2^)	−0.177	<0.001	−0.138	<0.001
Urine protein (g/day)	0.014	0.961	0.234	0.383
ALT (U/L)	−0.131	0.007	−0.118	0.008
AST (U/L)	−0.104	0.195	−0.113	0.122
TP (g/L)	0.112	0.161	0.089	0.222
ALB (g/L)	0.046	0.673	−0.025	0.8
FBG (mmol/L)	0.786	0.002	1.171	<0.001
BUN (mmol/L)	0.925	<0.001	0.772	<0.001
Scr (umol/L)	0.038	<0.001	0.033	<0.001
UA (umol/L)	−0.004	0.639	−0.008	0.275
TC (mmol/L)	−0.876	0.047	−0.307	0.447
TG (mmol/L)	−1.099	0.01	−0.449	0.253
HDL (mmol/L)	−2.667	0.173	−0.303	0.866
LDL (mmol/L)	−0.573	0.256	−0.299	0.517
Ca (mmol/L)	−3.689	0.484	−2.361	0.624
P (mmol/L)	3.626	0.27	6.657	0.026
K (mmol/L)	4.934	0.004	4.597	0.001
Na (mmol/L)	0.112	0.734	−0.253	0.4
Mg (mmol/L)	29.447	<0.001	18.726	0.012
Hb (g/L)	−0.191	<0.001	−0.185	<0.001
PLT (10^9^/L)	−0.041	<0.001	0.25	0.881
HCY (umol/L)	0.02	0.782	−0.023	0.734
PT (s)	−0.06	0.588	−0.089	0.374
APTT (s)	−0.118	0.406	−0.1	0.437
Fbg (g/L)	0.646	0.264	1.182	0.025
D-Dimer (ug/mL)	−0.667	0.379	−0.472	0.496
CRP				
Tertile 0	-	-	-	-
Tertile 1 (range) (0.048–0.09775)	2.473	0.311	3.334	0.134
Tertile 2 (range) (0.09776–0.1)	3.356	0.130	4.061	0.044
Tertile 3 (range) (0.1001–0.204)	1.842	0.503	2.225	0.375
Tertile 4 (range) (0.20401–2.548)	4.186	0.088	4.376	0.051
BMI				
BMI < 18.5	-	-	-	-
18.5 ≤ BMI < 24	−0.702	0.907	1.033	0.851
24 ≤ BMI < 28	2.796	0.639	3.609	0.508
28 ≤ BMI < 30	−0.600	0.924	0.923	0.872
30 ≤ BMI < 40	−4.323	0.491	−2.708	0.637
CKD stage				
1	-	-	-	-
2	3.795	0.077	1.614	0.414
3	8.729	<0.001	4.927	0.007
4	12.186	<0.001	8.396	<0.001
5	20.784	<0.001	18.123	<0.001
Medications				
ACEI/ARB (%)	−4.651	0.004	−4.983	0.001
Antiplatelet (%)	1.24	0.498	0.25	0.881
Statins (%)	1.94	0.216	2.89	0.043
Immunosuppressant (%)	−7.292	<0.001	−5.388	0.001
glucocorticoid (%)	−8.779	<0.001	−7.097	<0.001

Abbreviations: ACEI: angiotensin-converting enzyme inhibitor; AIx: arterial reflection wave enhancement index; AIx@75: AIx value at a heart rate of 75 bpm; ALT: alanine aminotransferase; APTT: plasma-activated partial thromboplastin time; ARB: angiotensin receptor blocker; AST: aspartate aminotransferase; ALB: albumin; BMI: body mass index; BUN: blood urea nitrogen; CHD: coronary heart disease; CRP: C-reactive protein; D-Dimer: D-dimer; eGFR: estimated glomerular filtration rate; FIB: plasma fibrinogen; FBG: fasting blood glucose; Hb: hemoglobin; HCY homocysteine; HDL: high-density lipoprotein; LDL: low-density lipoprotein; MAP: mean arterial pressure. PLT: platelet count; PT: prothrombin time; Scr: blood creatinine; TC: total cholesterol; TG: triglycerides; UA: blood uric acid.

**Table 4:** Multivariate linear analysis of AIX@75 correlation factors in CKD patients.

Model	β	95% CI	*p*
Model 1			
Female	9.583	6.746, 12.419	<0.001
Age (years)	0.512	0.418, 0.606	<0.001
MAP (mmHg)	0.266	0.170, 0.363	<0.001
Smoking	2.935	0.004, 5.867	0.05
Model 2			
eGFR (mL/min/1.73 m^2^)	−0.103	−0.148, −0.059	<0.001
Hb (g/L)	−0.101	−0.170, −0.031	0.005
Model 3			
Female	8.779	6.141, 11.417	<0.001
Age (years)	0.448	0.347, 0.548	<0.001
MAP (mmHg)	0.245	0.148, 0.343	<0.001
eGFR	−0.059	−0.096, −0.022	0.002
Model 4			
Female	8.912	6.273, 11.551	<0.001
Age (years)	0.425	0.321, 0.528	<0.001
MAP (mmHg)	0.228	0.130, 0.327	<0.001
eGFR	−0.049	−0.088, −0.011	0.013
Cause of disease			
Immune nephropathy	-	-	-
Metabolic nephropathy	3.093	−0.061, 6.246	0.055
Metabolic and immune combined nephropathy	6.539	0.595, 12.482	0.031
Unidentified	0.763	−2.901, 4.426	0.683
Model 5			
Female	8.926	6.291, 11.562	<0.001
Age (years)	0.485	0.390, 0.580	<0.001
MAP (mmHg)	0.255	0.159, 0.350	<0.001
ACEI/ARB	−4.466	−6.963, −1.969	<0.001
Glucocorticoid	−3.163	−6.143, −0.183	0.038

Note: Model 1 included female sex, age, BMI, MAP, diabetes mellitus, CHD, and intrarenal arteriosclerosis; Model 2 included eGFR, ALT, AST, P, K, Mg, Hb, Fbg, Hb, TC, TG, and HDL; Model 3: Models 1 and 2 were included; Model 4: Model 3 plus etiology (immune nephropathy, metabolic nephropathy, metabolic and immune combined nephropathy, unidentified); Model 5: Model 4 plus medication history (ACEI/ARB, antiplatelet, statins, immunosuppressant, glucocorticoid). The results of the multivariate linear analysis are summarized in Table 4. Abbreviations: ACEI: angiotensin-converting enzyme inhibitor; ARB: angiotensin receptor blocker; eGFR: estimated glomerular filtration rate; MAP: mean arterial pressure.

## Data Availability

Some or all datasets generated during and/or analyzed during the current study are not publicly available but are available from the corresponding author upon reasonable request.
